# Management of Open Tibial Diaphyseal Fractures by Limb Reconstruction System As Primary and Definitive Treatment: A Prospective Cohort Study

**DOI:** 10.7759/cureus.39774

**Published:** 2023-05-31

**Authors:** Jayesh K Soni, Shreepad Kulkarni, Gireesh Khodnapur, Rajkumar Bagewadi, Santosh S Nandi

**Affiliations:** 1 Orthopaedic Surgery, Shri B.M. Patil Medical College Hospital and Research Centre, BLDE (Bijapur Lingayat District Educational Association) University, Vijayapura, IND

**Keywords:** modified anderson’s and hutchin’s criteria, complications, union rate, gustilo-anderson classification, limb reconstructive system, open tibial diaphyseal fractures

## Abstract

Background

One of the most frequent long bone fractures that most orthopaedic surgeons see is a tibial diaphyseal fracture. The tibia has more open fractures than any other major long bone because it is covered by skin for the majority of its length. The best course of therapy is still up for debate due to the high occurrence of comorbidities linked to these fractures.

Materials and methods

In this prospective study, 30 patients who met the inclusion criteria were admitted to the Department of Orthopaedics of Shri B. M. Patil Medical College Hospital and Research Centre, Vijayapura, Karnataka, India. The period of study was from January 2021 to May 2022. The patients were followed up for a period of six months. Longer follow-up was required for some patients.

Results

In our study, there were 26 (86.7%) male and four (13.3%) female patients. The mode of injury was road traffic accidents in all cases. The functional outcomes obtained using the modified Anderson and Hutchinson's criteria were good results in 22 (73.3%), moderate results in five (16.7%), and poor results in three (10%) of the study population. Pin tract infections (six cases; 20%) and shortening (eight cases; 26.7%) were the most frequent complications

Conclusion

Because of the ease of use, good fracture stability, adjustable geometry, light weight, reasonable price, and patient friendliness, the limb reconstruction system (LRS) provides an excellent alternative treatment option for treating compound fractures of the tibia.

## Introduction

Open tibial diaphyseal fractures are severe injuries that can lead to significant long-term disability if not treated appropriately. Such fractures often result from high-energy trauma, such as motor vehicle accidents or falls from a height. Treatment of these fractures can be challenging and requires a multidisciplinary approach. In recent years, technological advances have led to the development of new techniques for treating these fractures, including using limb reconstruction systems (LRS) [1].

LRS are external fixation devices that allow for the gradual correction of deformities and the restoration of limb length and alignment. These systems consist of external fixators, which are connected to the bone using pins or wires, and can be adjusted to provide precise control over the position and orientation of the bone fragments. The use of LRS has been shown to be effective in treating a wide range of fractures, including those of the tibia [2].

The primary objective of treating open tibial diaphyseal fractures is to achieve fracture union while minimizing the risk of infection. Treatment typically involves surgical debridement of the wound, followed by the stabilization of the fracture using internal or external fixation and wound coverage. The choice of treatment depends on the severity of the fracture and the presence of associated injuries [3].

While there is general agreement on the importance of early surgical intervention for open tibial diaphyseal fractures, there is an ongoing debate about the optimal timing of definitive fixation. Some surgeons advocate early definitive fixation, while others prefer to delay surgery until soft tissue injuries have healed. The optimal timing of surgery is critical because early fixation can promote fracture union and minimize the risk of complications, while delayed surgery can lead to prolonged hospital stays, increased morbidity and mortality, and decreased functional outcomes [4-5].

The use of LRS for the treatment of open tibial diaphyseal fractures has been gaining popularity in recent years, as these devices allow for the early stabilization of fractures while avoiding the risks associated with early definitive fixation. The LRS provides a stable construct that allows for the correction of deformities and the restoration of limb length and alignment [6].

Despite the growing popularity of LRS, there is limited research on their efficacy in the treatment of open tibial diaphyseal fractures. Previous studies have reported high union rates and low complication rates, but few studies have evaluated functional outcomes. This study aims to evaluate the functional outcomes of patients with open tibial diaphyseal fractures treated with LRS [7].

## Materials and methods

Study design and patient selection

This prospective cohort study was conducted at the Department of Orthopaedics of Shri B.M. Patil Medical College Hospital and Research Centre from January 2021 to May 2022, and included 30 patients diagnosed with open tibial diaphyseal fractures. Patients above 18 years with open tibial type II, IIIA and IIIB diaphyseal fractures as classified by Gustilo-Anderson classification [8] were included. Patients with closed fractures, open type I, IIIC fractures and segmental fractures of tibia, tibial fractures with intra-articular extension, and open tibial fractures associated with ipsilateral fracture femur (floating knee) were excluded.

**Figure 1:**
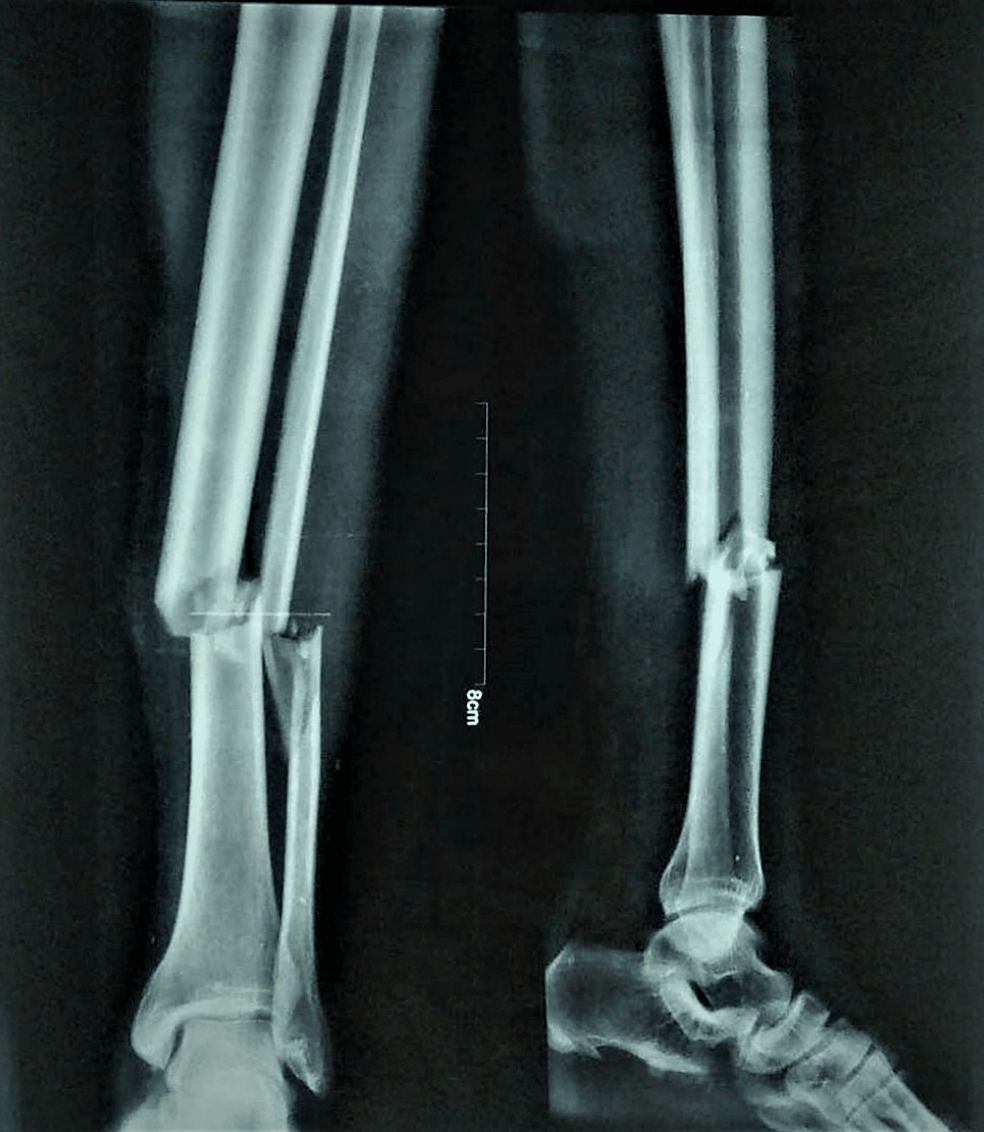
Preoperative X-ray (anteroposterior and lateral views)

All 30 patients got regular examinations and underwent detailed clinical evaluations about the mode of injury and previous treatments. A thorough examination was conducted to see whether the area around the tibia and its surroundings had any soft tissue, bone, tendon, or neurovascular injuries. Fractures were categorised using the Gustilo-Anderson classification [8] and standard anteroposterior (AP) and lateral roentgenographic images of the injured limb, including the distal and proximal tibia, were taken (Figure 1).

Patients who were not hemodynamically stable were stabilised first, and the mangled extremity severity score (MESS) was used to determine if an extremity could be saved. All necessary preoperative examinations were conducted on hemodynamically stable patients.

Surgical technique

**Figure 2:**
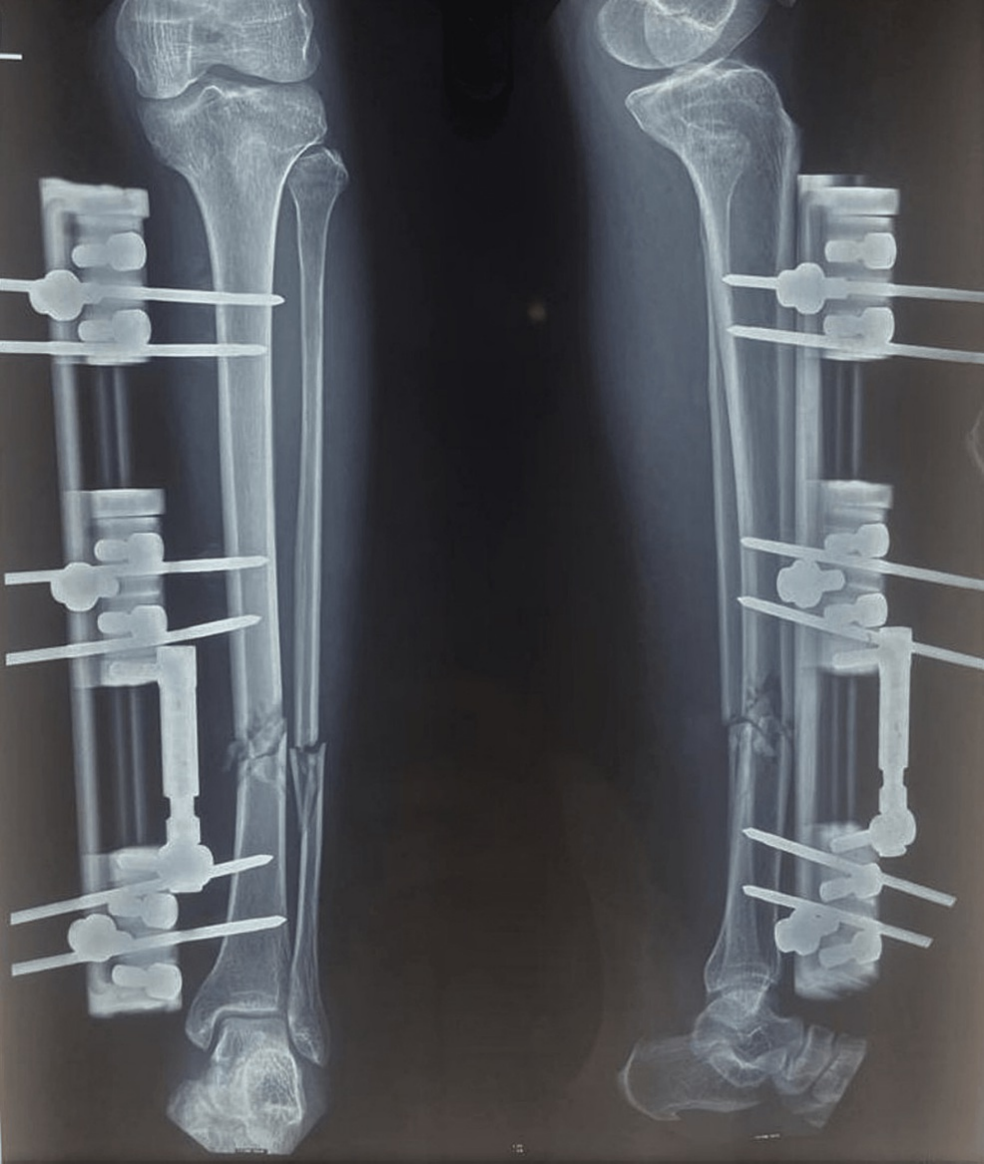
Immediate postoperative X-ray (anterioposterior and lateral view)

The LRS was implanted on the anteromedial surface of the tibia, preferable, in order to give adequate room for the desired soft tissue operation (Figure 2).

All dead necrotic tissues and free loose bone fragments were concurrently removed during surgical debridement. In cases of transverse and oblique fractures, acute docking was performed. Regular dressing, delayed primary closure, secondary closure, and split skin grafting were all used in wound care. In other instances, secondary intention led to wound healing. Intravenous (IV) antibiotics were administered until the wound healed, after which oral antibiotics were administered. In all patients, exercises for the quadriceps, hamstrings, and range of motion in the knee and ankle were started on the second postoperative day. Once there were radiological and clinical indicators of union, full weight bearing with a walker was initiated.

Outcome measures

**Figure 3:**
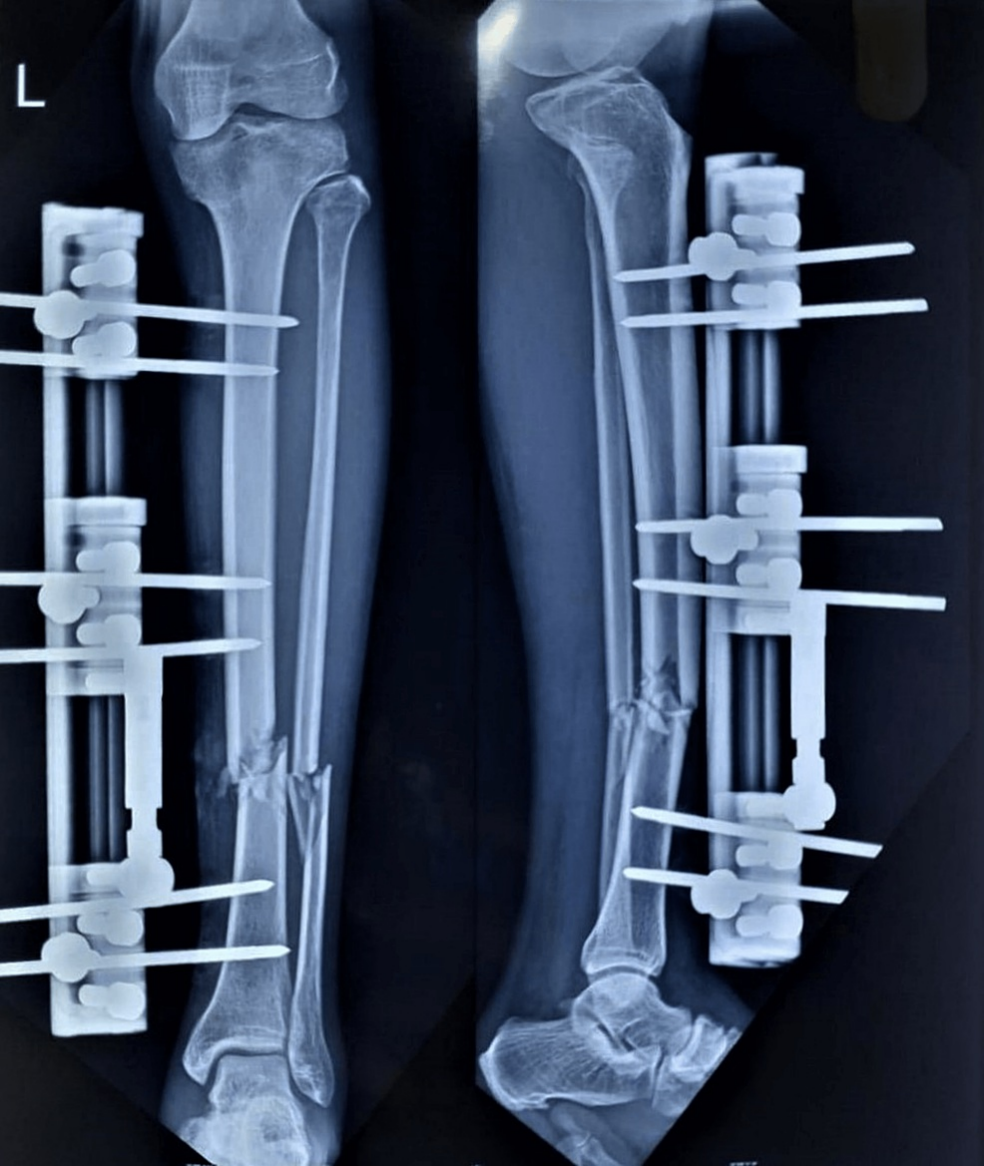
Six weeks postoperative X-ray (anteroposterior and lateral view)

Follow-up was conducted until the union was established clinically and radiologically at regular intervals of six weeks for six months (Figure 3).

Each follow-up visit included an evaluation of complications, including muscle contractures, joint dislocation, axial deviation, neurological or vascular insult, premature or delayed consolidation, refracture, and pin-site infection, which were then treated appropriately. A bridging callus was used radiologically to define healing. Clinically speaking, the union was defined as the lack of discomfort and mobility at the fracture site. If three of the four cortices exhibited bridging callus, the fracture was deemed to be united radiographically. The final assessment for functional outcome and results was done using modified Anderson and Hutchinson’s criteria (Table 1) [9]. 

**Table 1:** Modified Anderson and Hutchinson’s criteria

Results	Shortening	Grade of deformity in Angulation (Malunion)
Good	< 1 cm	< 5°varus / valgus, < 10° Anterior/Posterior, full range knee and ankle movement and painless walking, sitting cross-legged and squatting normal, no pain, no swelling, no tenderness
Moderate	1-2 cm	5-10° varus / valgus, 10-20° Anterior/Posterior, insignificant loss of motion at knee and ankle
Poor	> 2 cm	> 10° varus / valgus, > 20° Anterior/Posterior angulation, significant loss of movement at knee and ankle

## Results

In our study, the majority of patients were in the age group of 31-40 years with a mean age of 38.3 years. The oldest was 60 years old, while the youngest was 21 years old. The majority of participants were male (n=26; 86.7%) and females were four (13.3%), which may indicate that men engage in more outdoor activities. Road traffic accidents accounted for 30 out of 30 instances (100%) in our study. As per the Gustilo-Anderson classification, the maximum number of cases were type II patients (n=18; 60%). Types IIIA and IIIB included eight (26.7%) and four (13.3%) cases, respectively. There was 53.3% (n=16) right-sided prevalence and 46.7% (n=14) left-sided prevalence.

Soft tissue reconstruction was done in eight (26.7%) patients, seven (23.3%) had split skin grafts, and one (3.3%) had muscle pedicle flaps. Bone grafting was necessary in three (10%) cases with soft tissue reconstruction. The intact or joined fibula may prevent the compression of the fracture site; hence, one patient (3.3%) in our study needed a fibulotomy, two (6.7%) patients underwent secondary closure, while one (3.3%) patient received delayed secondary closure. 

One (3.3%) type IIIB and one type II fracture patient in our dataset had hypertension and were given antihypertensives. Human Actrapid insulin successfully treated one patient who had type II diabetes. Two (6.7%) patients had a history of asthma and were using a Deriphylin pill and salbutamol nebulizer. 

The average duration of hospital stay was 33 days, while maximum stay was 50 days and minimum 18 days. Pin tract infections and shortening were the most frequent complications. After receiving a culture and sensitivity report, six (20%) pin tract infections were treated with parenteral antibiotics. Eight (26.7%) cases of shortening were observed, with one (3.3%) case of equinus deformity. In one instance, a deep infection took place. In two (6.7%) cases, the union was delayed (Table 2).

**Table 2:** Complications

Complications	No. of patients
Pin tract infection	6
Shortening	8
Equinus deformity	1
Deep infection	1
Malunion	1
Delayed union	2

The modified Anderson and Hutchinson's criteria were used to determine the degree of deformity, limb length disparity and functional outcome. In our study, results were good in 22 (73.3%) patients, moderate in five (16.7%), and poor in three (10%) (Table 3).

**Table 3:** Results

Results	Number of patients	Percentage
Good	22	73.3
Moderate	5	16.7
Poor	3	10

## Discussion

Most of the patients were between 31 and 40 years of age. Our study's average age was 38.3 years old while the mean age in Thakur and Patankar's study was also 38 years [10]. Twenty-six (86.7%) patients in our study were males and the remaining four (13.3%) were female patients. Similarly, 83.5% of the participants were male in the study conducted by Thakur and Patankar and 16.5% were female [10].

In our study, all 30 (100%) injuries resulted from traffic-related incidents, while in the study by Antich-Adrover et al., 81.9% of cases in group A and 90% of cases in group B had injuries as a result of traffic accidents [11]. Road traffic accidents accounted for 87.3% of open fractures in the Thakur and Patankar series [10].

According to the Gustilo-Anderson classification, we included types II to IIIB in our study. The majority of the patients in our research belonged to the Gustilo-Anderson Type II (n=18 cases; 60%). The Type IIIA and Type IIIB groups came next, accounting for eight (26.7%) and four (13.3%) cases, respectively. There were 40 (50.63%) comminuted fractures, 27 (34.18%) transverse or short oblique fractures, and 12 (15.19%) spiral or long oblique fractures in the Thakur and Patankar series [10].

In our series, seven patients (23.3%) had split skin grafts, one patient (3.3%) had a muscle pedicle flap, one patient (3.3%) had delayed primary closure, and two patients (6.7%) had secondary closure. In Tornetta III et al.'s study, a skin graft or muscle flap was used to achieve tissue cover in each patient [12].

Bone grafting was done in three (10%) cases after radiography revealed non-union symptoms. In contrast, bone grafting was performed in 44 (60.3%) cases in Thakur and Patankar’s study [10].

In our research, eight patients (26.7%) experienced limb shortening and seven patients (23.3%) had pin tract infections, which were both frequent complications. With the exception of one case, all the pin tract infections were successfully treated with the appropriate IV antibiotics following culture and sensitivity testing. Poor nutrition, nosocomial infections, and inability to pay for medications were considered to be the main contributing factors to infection. In a study by Bhandari et al., one (3.3%) patient had deep infections [13]. In our research too, deep infections affected one (3.3%) patient.

## Conclusions

Early stabilisation of an open tibial fracture using LRS and immediate soft tissue covering led to excellent fracture union, low rates of complications, and good functional outcomes when compared to other treatment techniques. With a short duration of hospital stay and an early return to work, it is also economically beneficial. Also, overall morbidity is reduced, and overall patient satisfaction is noted.
